# Untangling the roles of RNA helicases in antiviral innate immunity

**DOI:** 10.1371/journal.ppat.1010072

**Published:** 2021-12-09

**Authors:** Morgane Baldaccini, Sébastien Pfeffer

**Affiliations:** Université de Strasbourg, Architecture et Réactivité de l’ARN, Institut de Biologie Moléculaire et Cellulaire du CNRS, Strasbourg, France; University of Alberta, CANADA

## Abstract

One of the first layers of protection that metazoans put in place to defend themselves against viruses rely on the use of proteins containing DExD/H-box helicase domains. These members of the duplex RNA–activated ATPase (DRA) family act as sensors of double-stranded RNA (dsRNA) molecules, a universal marker of viral infections. DRAs can be classified into 2 subgroups based on their mode of action: They can either act directly on the dsRNA, or they can trigger a signaling cascade. In the first group, the type III ribonuclease Dicer plays a key role to activate the antiviral RNA interference (RNAi) pathway by cleaving the viral dsRNA into small interfering RNAs (siRNAs). This represents the main innate antiviral immune mechanism in arthropods and nematodes. Even though Dicer is present and functional in mammals, the second group of DRAs, containing the RIG-I-like RNA helicases, appears to have functionally replaced RNAi and activate type I interferon (IFN) response upon dsRNA sensing. However, recent findings tend to blur the frontier between these 2 mechanisms, thereby highlighting the crucial and diverse roles played by RNA helicases in antiviral innate immunity. Here, we will review our current knowledge of the importance of these key proteins in viral infection, with a special focus on the interplay between the 2 main types of response that are activated by dsRNA.

## Introduction

To ward off viral infections, cells rely on a large variety of mechanisms that have been refined throughout evolution. During the initial response to an infection, the defense put in place will mainly consist in detecting the invading pathogen via the sensing of specific molecular patterns, which, in turn, will either trigger a signaling cascade or act directly on the detected molecule to hamper its function and/or degrade it. Different danger signals exist, but the presence of a foreign nucleic acid in the cell is one of the most prominent. Thus, the accumulation of DNA in the cytoplasm of eukaryotic cells, or of aberrant RNA molecules that do not possess the hallmarks of cellular RNAs, will immediately result in the onset of an innate immune response. Among the molecular features that are commonly associated with these pathogenic RNAs we can cite the presence of a 5′ triphosphate instead of a 5′ cap or of a double-stranded structure. In fact, double-stranded RNA (dsRNA) accumulates in cells infected by almost all viruses. While for RNA viruses, dsRNA is either generated during replication or by base pairing of single-stranded RNA regions within the genome, it can originate from convergent transcription for DNA viruses [1]. Therefore, dsRNA is a potent pathogen-associated molecular pattern (PAMP), which is recognized by both cytosolic and membranous receptors named pattern recognition receptors (PRRs). Upon dsRNA sensing, PRRs act in 2 different ways: either directly as effector molecules or indirectly as signal transducers. These receptors can be grouped into 3 different families: the Toll-like receptors (TLRs), the cytosolic NOD-like receptors (NLRs), and the cytosolic RIG-I-like receptors (RLRs) [2], which will be the subject of this review.

RLRs belong to a class of helicase-containing proteins able to sense nucleic acid to induce an antiviral response. They use ATP to bind to and/or unwind nucleic acids, thereby altering ribonucleoprotein complexes. Structural and sequence features that have been observed in archaea, bacteria, and eukaryotic cells allowed to classify RNA helicases into different superfamilies (SFs) [3]. Only SF1 and SF2 include helicases containing 2 bacterial RecA-like fold domains. The DExD/H-box helicase proteins family we are interested in are found within the SF2 subgroup [3]. This family is composed of RNA helicases involved in all aspects of RNA metabolism but also in antiviral defense [4,5]. These proteins can either unwind RNA upon ATP binding and hydrolysis or clamp around RNA to act as a platform to recruit other proteins [6].

Within the DExD/H-box helicases family, the subgroup of duplex RNA–activated ATPases (DRAs) is composed of SF2 helicases activated by dsRNA [7]. DRAs share the same activation mechanism: upon dsRNA binding, they undergo conformational changes to become catalytically active [8]. However, due to significant differences in their protein domain organization and their functions [6,8], we can distinguish signaling DRAs (sDRAs) and catalytic DRAs (cDRAs). These proteins include RLRs and Dicer proteins, which share a similar helicase domain [7,8] and participate in 2 main antiviral pathways, namely the RNA interference (RNAi) and the type I interferon (IFN) response. Here, we will focus on metazoan DRAs and more specifically on the 3 mammalian RLRs (RIG-I, MDA5, and LGP2), the mammalian Dicer, the 2 *Drosophila melanogaster* Dicers (dmDicer-1 and dmDicer-2), the *Caenorhabditis elegans* Dicer (ceDicer), and the 3 *C*. *elegans* Dicer-related helicases (DRH-1 to 3) [7]. We will discuss the role of DRAs as direct effectors of the antiviral response, before reviewing their roles as signaling molecules. Finally, we will elaborate on the noncanonical roles played by DRAs during viral infection in the light of recently published data and will comment on the possible crosstalk that can exist between pathways.

### RNA helicases as direct effectors of the antiviral response

The best example of helicases acting directly on viral dsRNA upon sensing is without any doubt the case of Dicer proteins, which are the primary effectors of antiviral RNAi. This ancestral defense mechanism is the main antiviral system in nematodes and insects [9,10], while in mammals, this role is fulfilled by type I IFN response [11]. However, as we will see later, RNAi has been shown to play a role in the fight against viruses in mammalian cells, and an IFN-like antiviral response in invertebrates has been described [12–15], further blurring the boundaries between these 2 major innate immunity mechanisms.

With the notable exception of *Saccharomyces cerevirisiae* [16], Dicer is conserved in most eukaryotes. This enzyme is involved in the microRNA (miRNA) pathway and in RNAi through the generation of small interfering RNA (siRNA) [17]. The origin of Dicer functional diversity can be found in early branching metazoans [18]. Some organisms, such as *Drosophila melanogaster*, present a duplication of the *dicer* gene, thus splitting the proto-function in 2. One protein (dmDicer-1) is involved in the miRNA pathway, whereas the other one (dmDicer-2) is responsible of both exogenous and endogenous long dsRNA processing [9]. Phylogenetically, dmDicer-2 is closer to the common ancestral RNase III than dmDicer-1 [18]. Conversely, the nematode *Caenorhabditis elegans* genome encodes one single Dicer (ceDicer), which is very similar to the proto-Dicer as it carries both functions in miRNA and siRNA pathways [19]. Similarly, only one Dicer (hsDicer), involved in the miRNA pathway, is present in humans [20]. The capacity of hsDicer to perform efficient antiviral RNAi is still a matter of debate [14,21].

**Fig 1:**
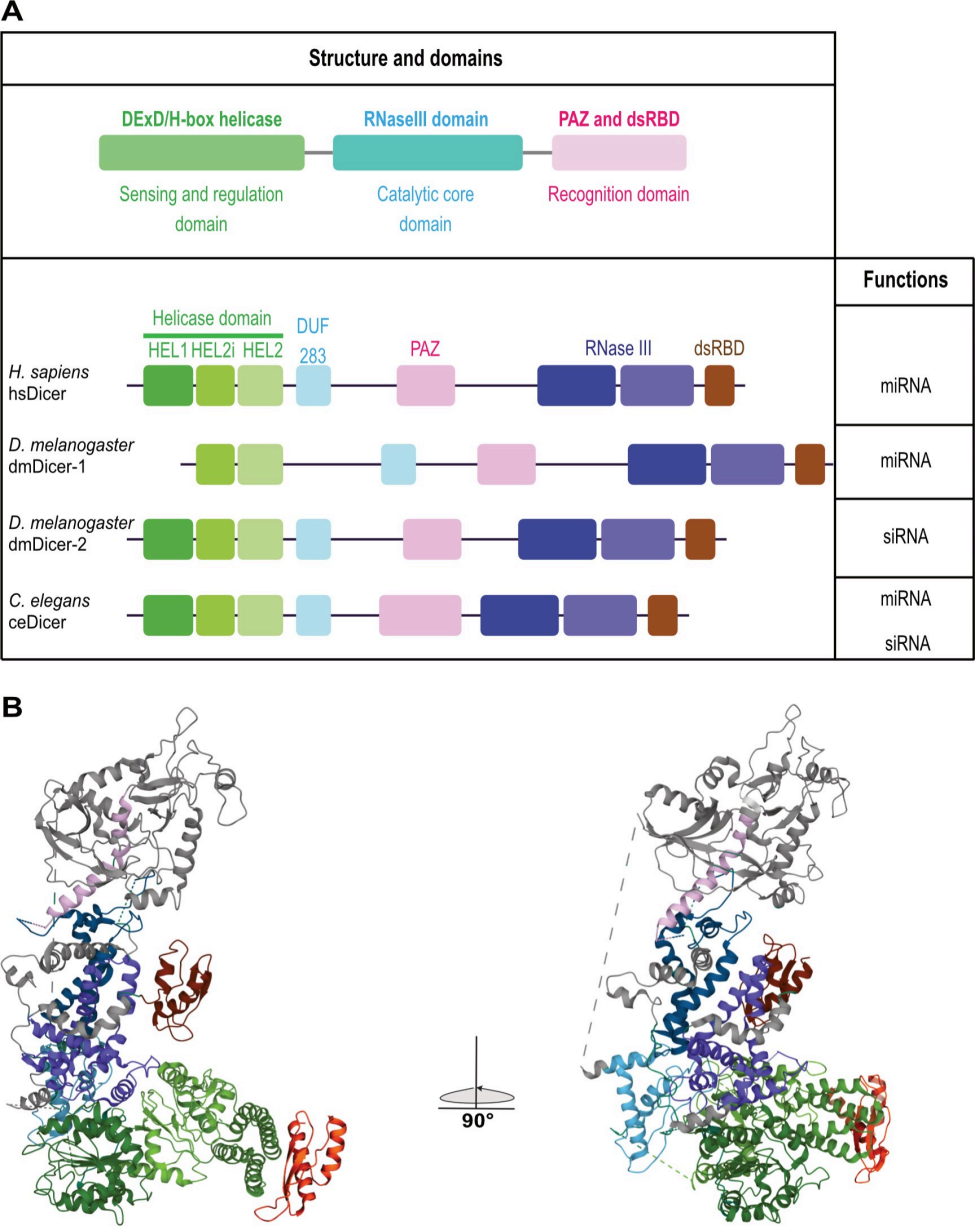
Domains organization and functions of Dicer proteins in metazoans. **(A)** Dicer carries a DExD/H-box helicase, which is divided into 3 subdomains: HEL1, HEL2i, and HEL2. The domain of unknown function (DUF283) is regulating pre-miRNA binding and the PAZ domain is involved in the recognition of the 3′-overhanged extremities. The catalytic core is composed of 2 RNase III domains that each processes one strand of the duplex. Finally, the terminal dsRBD is involved in the binding of the dsRNA minor groove. **(B)** Position of the different domains in the tridimensional structure of human Dicer in complex with TRBP determined by cryo-EM [22]. The color code used for the domains is the same as in A. TRBP is in orange (adapted from PDB structure n°5ZAK). cryo-EM, cryo-electron microscopy; dsRBD, dsRNA-binding domain; miRNA, microRNA; PAZ, Piwi–Argonaute–Zwille; siRNA, small interfering RNA; TRBP, TAR RNA-binding protein.

All these Dicer proteins share a similar organization in different domains, some of which are conserved with the bacterial RNase III [22] (Fig 1A). From the N-terminal part, there is a DExD/H-Box helicase split into 3 subdomains HEL1, HEL2i, and HEL2. Then, there is a domain of unknown function DUF283, which was computationally predicted [23] and recently structurally defined [22] as a dsRNA-binding domain (dsRBD). It is followed by an α-helix connector, Piwi–Argonaute–Zwille (PAZ) and Platform domains involved in the recognition of RNA with 3′-overhanging extremities [24]. Finally, toward the carboxyl-terminal part, there are a conserved tandem RNase III domain (IIIa and IIIb) that forms an intramolecular dimer to cleave RNA substrates [25] and a dsRBD able to bind the minor groove of dsRNA [26,27]. One hypothesis is that the helicase domain is involved in auto-inhibiting the proper function of Dicer. In *Drosophila*, the 2 Dicers differ by their helicase domain [28]. As opposed to dmDicer-2, dmDicer-1 contains only a truncated version of the helicase with just a HELICc motif (Fig 1A). By alignment of metazoans helicases, it can be observed that dmDicer-1 is the least conserved among them with divergences in the ATP-binding and in the intramolecular interaction motives [29]. Conversely, the helicase domain of dmDicer-2 is more conserved and is involved in its processivity as it hydrolyzes the ATP necessary for its translocation along the dsRNA [29]. The ceDicer also hydrolyzes ATP to translocate, whereas the hsDicer protein does not require ATP hydrolysis and seems to be closer to dmDicer-1 helicase [19,22]. As such, the helicase domain has an auto-inhibitory effect on hsDicer activity [30]. In addition, it is involved in the recruitment of regulatory co-factors such as TAR RNA-binding protein (TRBP), protein activator of the interferon-induced protein kinase (PACT), and ATP-dependent RNA Helicase A (DHX9) [31–34].

Recently, the determination of the 3D structure of hsDicer allowed novel features of its helicase domain to be uncovered. Cryo-electron microscopy revealed that hsDicer adopts an L-shaped structure where the helicase domain is located in the shorter arm [22,35,36] (Fig 1B). The 3 subdomains of the DExD/H-box helicase form a C-shaped structure in which HEL1 is at the junction between the 2 parts of the “L” and interacts with DUF283 and RNaseIIIb. HEL2 is at the center of the “C-shaped” structure, while HEL2i is at the arm tip and is involved in mediating the interaction with TRBP [31,32]. Finally, an α-helix connects HEL1 and HEL2 forming a pincer-like motif with a flexible HEL1 that can easily move around substrates (Fig 1B). Interestingly, dmDicer-2 cryo-electron microscopy structure resembles the hsDicer “L-shaped” structure [37].

**Fig 2:**
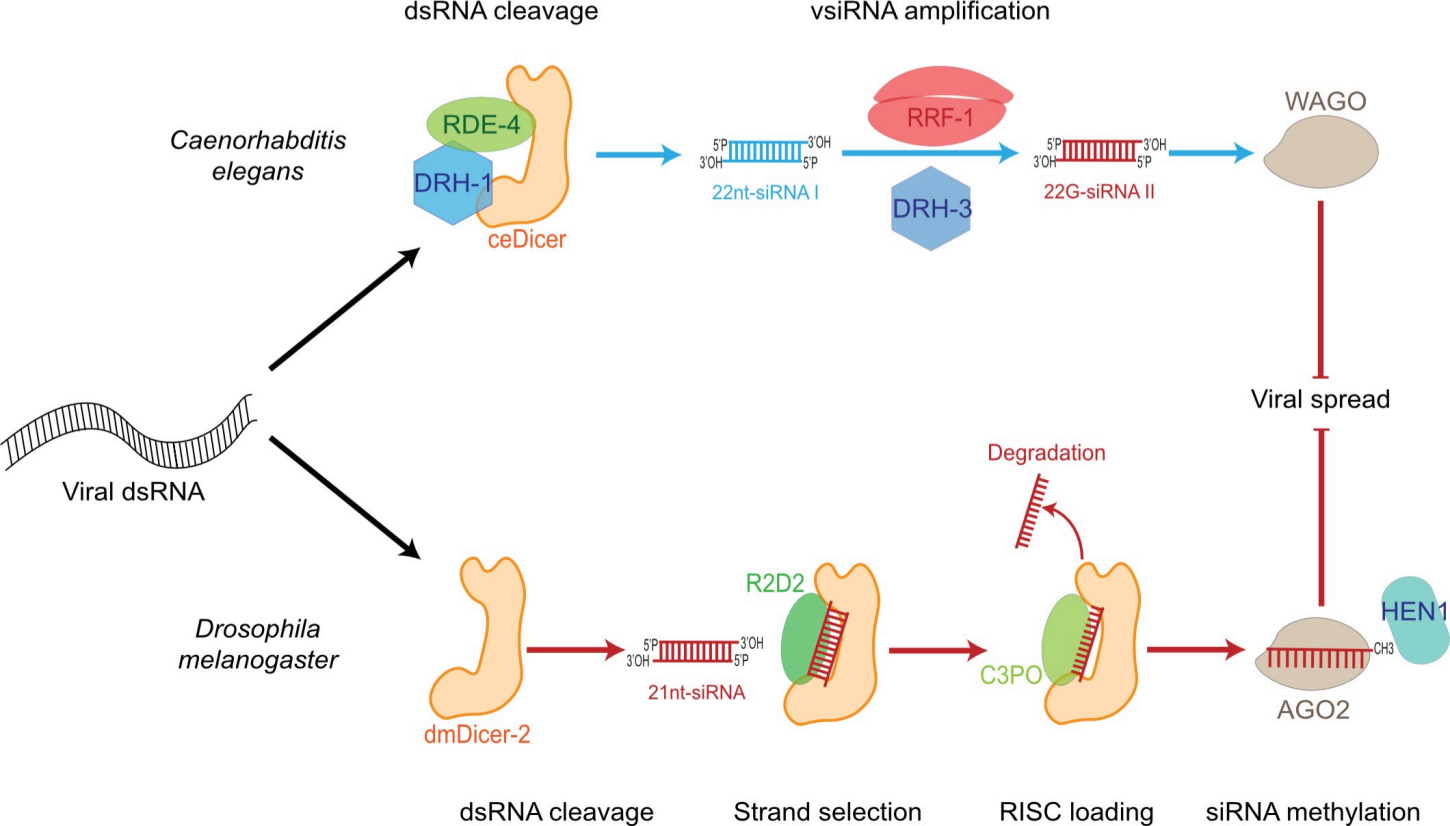
Antiviral RNAi pathways in *Caenorhabditis elegans* and *Drosophila melanogaster*. Upon viral infection, dsRNA triggers RNAi after its recognition by Dicer. In *C*. *elegans*, Dicer helped by the dsRBP RDE-4 and another DExD/H-box helicase, DRH-1, recognizes the dsRNA and processes it into primary 22 nt siRNA duplexes. These duplexes serve as a template for the generation of secondary 22G-siRNA by the polymerase RRF-1 and a third helicase called DRH-3. They are finally loaded into an Argonaute protein, WAGO. In *D*. *melanogaster*, Dicer-2 recognizes and cleaves the viral dsRNA into 21nt-siRNA duplexes. Strand selection occurs with the help of the dsRBP R2D2, and the passenger strand is sliced by Ago2 before being degraded by the nuclease C3PO. The guide strand is then 2′-O-methylated at the 3′ end by HEN1 to be stabilized. For both worms and flies, the loaded Argonaute protein can then cleave viral complementary sequences, resulting in the antiviral state. dsRNA, double-stranded RNA; RNAi, RNA interference; siRNA, small interfering RNA; vsiRNA, virus-derived small interfering RNA.

In both *C*. *elegans* and *D*. *melanogaster*, Dicer proteins are involved in antiviral RNAi. Despite several differences in the RNAi pathway of the 2 organisms, the key steps are conserved (Fig 2). In the worm, Dicer is involved in viral siRNA production that, in turn, causes a decrease in viral load [10,38]. ceDicer triggers RNAi against vesicular stomatitis virus (VSV) [10,38]. These results were later confirmed with the Orsay virus (OrV), which is a natural pathogen of *C*. *elegans* [39–41]. The viral dsRNA is recognized by a complex composed of ceDicer, the RNA helicase DRH-1, and the Argonaute protein RDE-1 [42–44]. The latter is not efficient in RNA silencing, even though it can target specific RNAs. However, silencing can be amplified by secondary siRNAs, or 22G-RNAs, which are generated by an RNA-dependent RNA polymerase RRF-1 and another RNA helicase, DRH-3 [45–47]. In somatic cells, these secondary siRNAs are then loaded in another Argonaute protein, WAGO [46,48]. This amplification system is common to all siRNA pathways in *C*. *elegans* and allows to increase both the siRNA pool and targeted sequences on RNAs [44]. A point mutation in ceDicer helicase domain dramatically decreases virus-derived small interfering RNA (vsiRNA) and endogenous small interfering RNA (endosiRNA) production but has no consequences on exosiRNA production [49–51]. Moreover, this helicase domain is essential for siRNA production from internal regions of blunt-ended dsRNA [52].

In the fly *D*. *melanogaster*, dmDicer-2 is involved in the recognition and cleavage of endogenous dsRNA, exogenous dsRNA, and viral dsRNA [15]. Since dmDicer-2 is not an essential gene, it could be inactivated to show that RNAi is indeed a defense mechanism against many (+) ssRNA viruses such as Flock house virus (FHV), *Drosophila* C virus (DCV), Cricket paralysis virus, and Sindbis virus (SINV) [53–57]. In the viral dsRNA maturation process, the D isoform of the Loquacious protein (Loqs-PD) is dispensable, whereas R2D2 is required for vsiRNA loading into Ago2 [58]. In vitro, dmDicer-2 was shown to act independently from other factors to process long dsRNA thanks to its helicase domain [59]. The generated siRNA duplexes are then transferred from dmDicer-2 to another dsRBP, R2D2, to bring the duplexes to Ago2 and allow the formation of a pre-RISC complex. To be active, Ago2 cleaves the passenger strand, which is then degraded by the C3PO nuclease [60]. The guide strand is 2′-O-methylated at its 3′ terminal nucleotide to be stabilized and guides Ago2 to target RNAs, which are sliced and degraded [15]. The helicase domain in dmDicer-2 is multifunctional allowing substrate recognition, cleavage efficiency, and discrimination of dsRNA extremities. How can dmDicer-2 discriminate between viral dsRNA and other dsRNA molecules? One hypothesis is that viral dsRNA carries specific structures recognized by Dicer, similar to what happens with mammalian RLRs. Indeed, the helicase domain of dmDicer-2 is necessary for antiviral RNAi and permits the recognition of noncanonical extremities on dsRNA [51]. This domain can be found in 2 states, one of them allowing it to widen its range of recognizable dsRNA extremities following conformational change induced by ATP binding [61].

### RNA helicases with signaling antiviral activities

**Fig 3:**
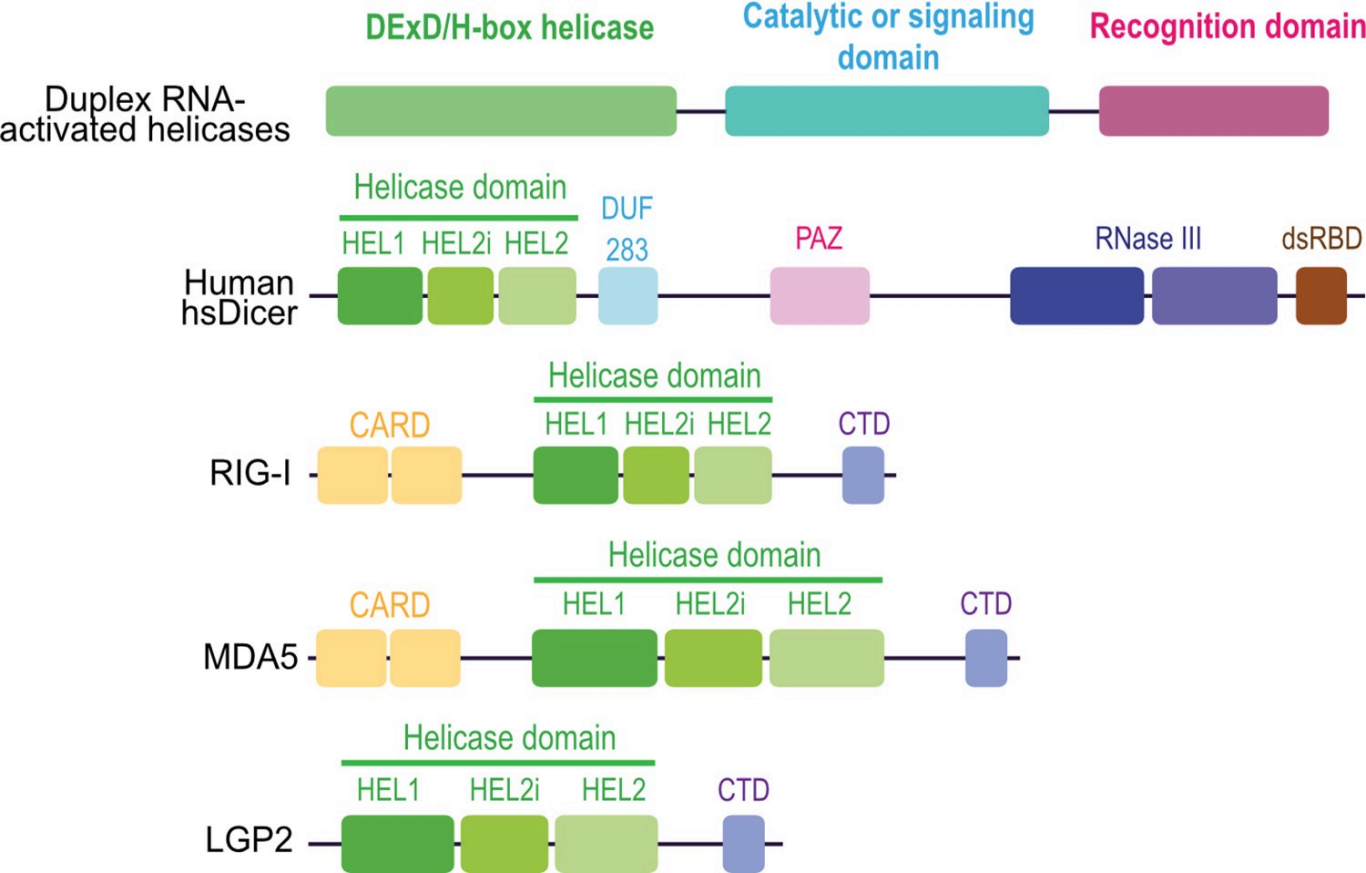
Domains organization of RLRs. RLRs are directly involved in the IFN-I response upon viral infection. RIG-I and MDA5 can activate the mitochondrial adaptor protein MAVS via their N-terminal CARD domains. Their central DExD/H-box helicase domain shares the same organization as the Dicer one and, together with the CTD, is involved in dsRNA recognition. LGP2 lacks the signaling CARD domains but possesses the whole helicase domain and the CTD. CARD, caspase activation and recruitment domain; CTD, carboxyl-terminal domain; IFN, interferon; MAVS, mitochondrial antiviral signaling adaptator; MDA5, melanoma differentiation–associated gene 5; RIG-I, retinoic acid–inducible gene I; RLR, RIG-I-like receptor; LGP2, laboratory of genetics and physiology 2.

As opposed to the situation in arthropods and nematodes, the main antiviral response in mammals is based on type I IFN. At the heart of this pathway are 3 cytosolic nucleic acid sensors called RLRs. Retinoic acid–inducible gene I (RIG-I or DDX58) recognizes 5′ di- or tri-phosphorylated dsRNA or ssRNA [62,63]. Melanoma differentiation–associated gene 5 (MDA5 or Helicard or IFIH1) recognizes long dsRNA [62,63]. Laboratory of genetics and physiology 2 (LGP2 or DHX58) modulates the functions of the 2 other RLRs instead of acting as a signal transducer (reviewed in [64]). RIG-I and MDA5 are composed of 4 domains (Fig 3) [65,66]: 2 N-terminal caspase activation and recruitment domains (CARDs), a central DExD/H-box helicase domain, and a pincer domain that connects the helicase to the regulatory carboxyl-terminal domain (CTD). The latter is essential for the recognition of the RNA substrate and autoinhibition of the protein when not RNA-bound. LGP2 is highly similar to RIG-I and MDA5 except it does not have CARD domains (Fig 3). Phylogenetic studies between all known PRRs show that the helicase domain of Dicer and RLRs are close in terms of sequence and structure [67]. Both belong to SF2 helicases and share some specificities that put them in the subclass of DRAs.

**Fig 4:**
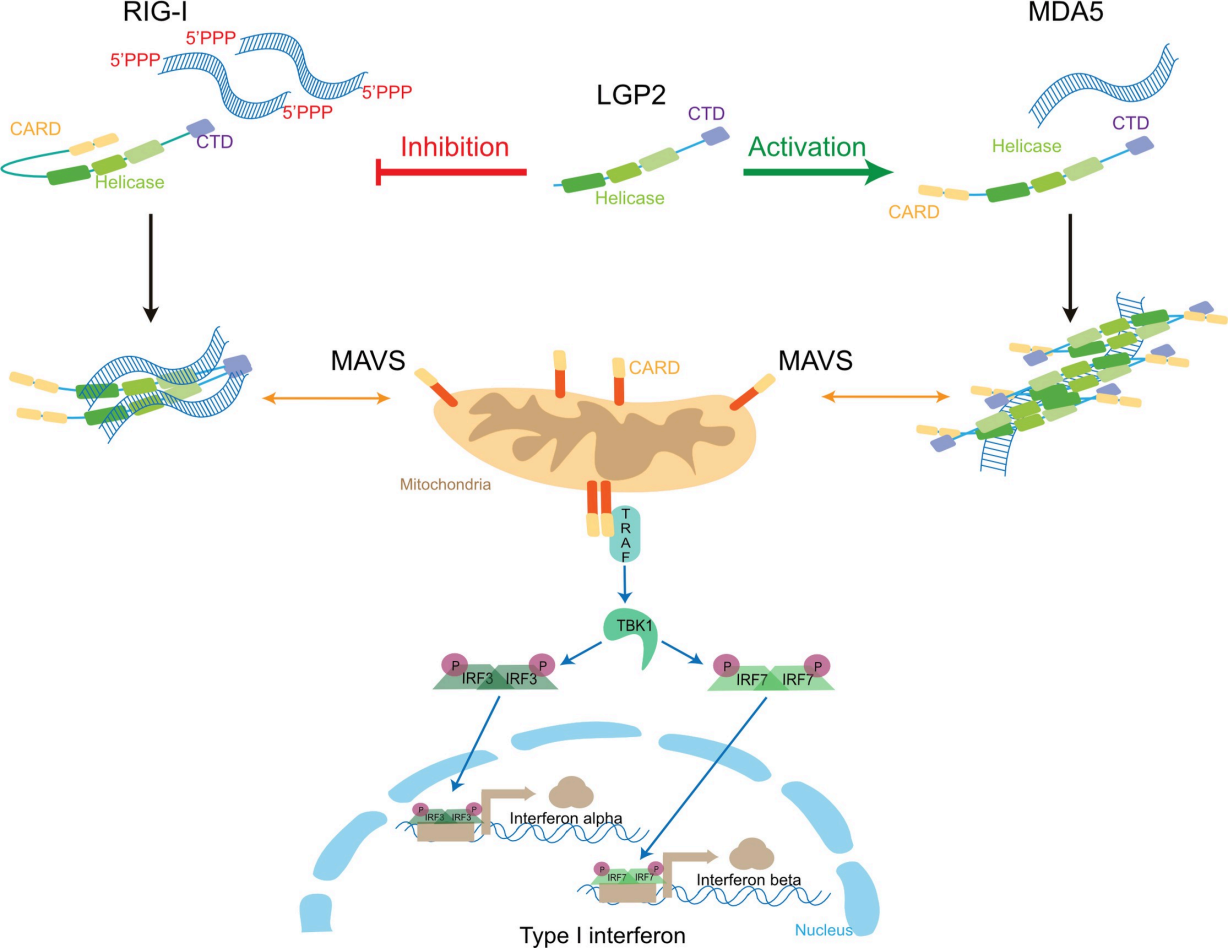
Mode of action of RLRs upon viral infection. Both dsRNA replication intermediates and 5′ terminal motifs can be recognized by cytosolic RLRs. RIG-I detects 5′ di- or tri-phosphorylated dsRNA via its CTD and helicase domains whereas MDA5 recognizes long dsRNA structures. Upon dsRNA binding, RIG-I opens and homodimerizes to mediate interactions with MAVS CARD domains at the surface of mitochondria. By contrast, MDA5 constantly shifts between open and close conformations and when it is activated, it polymerizes along the dsRNA forming helicoidal filament to expose its CARD domains and activate MAVS. Once activated, MAVS aggregates and triggers a signaling cascade through the TRAF protein that activates the TBK1 kinase. The latter then phosphorylates the cytosolic transcription factors, IRF3 and IRF7, thereby allowing their dimerization and their translocation into the nucleus where they activate the transcription of IFN-I genes. CARD, caspase activation and recruitment domain; CTD, carboxyl-terminal domain; dsRNA, double-stranded RNA; IFN, interferon; MAVS, mitochondrial antiviral signaling adaptator; MDA5, melanoma differentiation–associated gene 5; RIG-I, retinoic acid–inducible gene I; RLR, RIG-I-like receptor.

Like Dicer, RIG-I has a C-shaped DExD/H-box helicase domain composed of 3 subdomains: HEL1, HEL2i, and HEL2. HEL1 and HEL2 form the core helicase with dsRNA binding and ATPase functions. A flexible linker (HEL2i) with an autoregulatory role links these 2 domains [68]. The helicase and the CTD are involved in dsRNA and 5′ PPP-RNA recognition required for CARD-mediated signaling [68]. The CTD (also called repressor domain) together with the helicase domain form a ring around dsRNA adopting a compact conformation that, in absence of RNA substrates, is flexible and extended [68]. RIG-I can translocate along dsRNA (like dmDicer-2) but, as all helicases in the DRAs subclass, it cannot unwind the duplexes [69]. Under physiological conditions, RIG-I persists in the cytosol in an auto-inhibitory closed state where the tandem CARD domains are in head-to-tail conformation and directly interact with HEL2i to form an inactive structure [70]. dsRNA binding leads to a conformational change and to the stabilization of RIG-I clamp around dsRNA [71,72]. An activation model was proposed where dsRNA first binds the CTD, which then brings together several dsRNA molecules near the helicase domain. This increased dsRNA concentration permits the cooperative fixation of dsRNA and ATP on the helicase, thereby freeing the CARD domains that mask the dsRBD and the K63 polyubiquitination site [70] (Fig 4). ATP hydrolysis enhances RIG-I dissociation from dsRNA [66].

Although RIG-I and MDA5 share structural similarities, MDA5 is not activated by opening a closed conformation between its CARD and HEL2i domains. After dsRNA recognition in the cytosol, several MDA5 molecules gather and form helicoidal filaments along the dsRNA to coalesce into an active fibrillar form [73] (Fig 4). The formation of this MDA5 polar helix is allowed by cooperative assembly of their CTD. The contacts between filaments are supposedly mediated by HEL2i and CTD. ATP is involved in the disassembly of MDA5 oligomers, regulating dsRNA binding affinities in a concentration-dependent manner [73,74].

LGP2 lacks CARD in the N-terminal part, but it has a CTD and a DEAD/H-box helicase domain, which is highly conserved, showing a great selection pressure to maintain this domain [75]. Interestingly, LGP2 is a perfect chimera of the 2 other RLRs, with its helicase domain closer to the MDA5 one, while its CTD is closer to the RIG-I one. It can act both as an activator of MDA5 and an inhibitor of RIG-I [76]. Similar to MDA5, LGP2 can form helicoidal-like filaments in the cytosol [76]. Crystal structure obtained from chicken LGP2 indicates that it can adopt 2 conformations that are partially or fully closed [76]. These conformations are guided by ATP binding and hydrolysis and directed by the HEL2 helicase subdomain that is flexible and can orient its CTD [76]. The LGP2 structure after ATP hydrolysis is similar to the one adopted by RIG-I after activation [76].

Upon dsRNA recognition, both RIG-I and MDA5 interact with CARD-like domains of the mitochondrial antiviral signaling adaptator (MAVS) [77,78] (Fig 4). MAVS then activates a signal transduction cascade in 2 ways. On one hand, it interacts with TBK1 (TANK-binding kinase 1) and IKKε (IkB kinase ε), which will phosphorylate and induce translocation of a complex of transcription factors, IRF3 and IRF7 (IFN regulatory factor 3 and 7), which will activate IFN-I genes expression [77,78]. On the other hand, MAVS activates the NF-kB pathway leading to nuclear translocation of this transcription factor that induces expression of antiviral cytokines [77,78]. Through a molecular cascade involving the Janus kinase Signal transducer and activator of transcription (JAK-STAT) pathway, these cytokines finally activate hundreds of ISGs to prevent viral spreading [78–80].

Despite their similarities, RIG-I and MDA5 have nonredundant recognition capacities upon viral infection. Since they are activated by different substrates, they are not necessarily induced by the same viruses [63,66,81,82]. Blunt-ended dsRNA, 5′di or tri P, uncapped or 2′O-unmethylated 5′ extremities are RIG-I substrates. It is activated by rhabdoviruses (VSV), paramyxoviruses, orthomyxoviruses, filoviruses (Ebola), Epstein–Barr virus (EBV), hepatitis C virus (HCV), Japanese encephalitis virus, Zika virus (ZIKV), and Dengue virus (DENV) [66]. Interestingly, host RNAs can also be recognized as PAMPs by RIG-I [66]. For instance, upon infection by DNA viruses, such as Herpes simplex virus type I (HSV-1) and Kaposi sarcoma–associated herpesvirus (KSHV), host-encoded small noncoding RNAs can be recognized by RIG-I [83,84]. Rather, MDA5 is involved in the recognition of the dsRNA replication intermediate of picornaviruses [85]. MDA5 is also able to detect dsRNA from HCV, norovirus, encephalomyocarditis virus (EMCV), hepatitis B virus (HBV), herpes simplex virus, avian Influenza virus H5N1, and hepatitis D virus [86–92]. In some cases, such as infection with flaviviruses and reoviruses, both RIG-I and MDA5 seem to be recruited to trigger an IFN-I response [82].

### Noncanonical and interdependent actions of RNA helicases

In addition to performing the abovementioned activities, RNA helicases can also function in an uncommon manner. Thus, RLRs can have direct antiviral activities and Dicer proteins can exert signaling functions. In addition, such as in the case of mammalian Dicer, their role in antiviral RNAi may depend on additional factors as indicated below.

**Fig 5:**
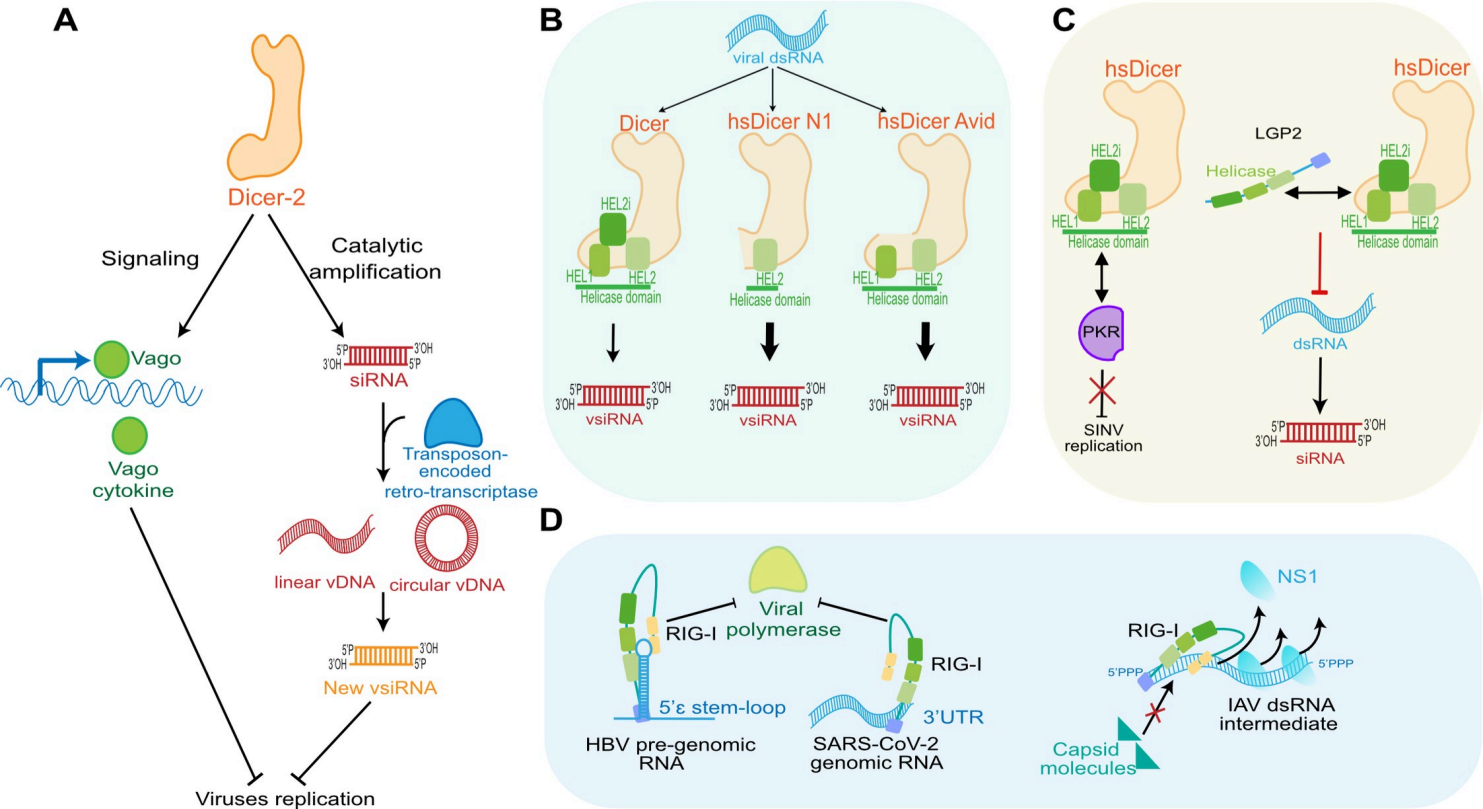
Noncanonical functions of helicases during viral infection. **(A)** In insects, Dicer-2 is involved in 2 antiviral pathways in addition to its canonical role in RNAi. First, its helicase domain is necessary to induce the transcription of Vago, a *Drosophila* cytokine. Vago inhibits viral replication through a yet to be defined mechanism. Second, in *Drosophila* and mosquitoes, Dicer-2-generated siRNAs can be used by a transposon-encoded retrotranscriptase to generate both linear and circular vDNA molecules. These vDNA can be a source of new vsiRNA duplexes, thereby amplifying the antiviral signal in the cell. **(B)** In the case of mammalian Dicer, its involvement in direct catalytic antiviral pathways remains debated. During viral infection of undifferentiated ESCs, Dicer is responsible for the production of vsiRNAs functionally involved in the blocking of viral replication. Artificial generation of a helicase-truncated form of Dicer (N1) allows to uncover antiviral RNAi functions in differentiated cells as well. The recent discovery in human ES cells of a naturally occurring isoform of Dicer called Avid, which lacks the HEL2i domain, provides some support to the existence of antiviral RNAi in humans. **(C)** In human cells, Dicer is also at the center of many interactions, which could modulate either its own or the interacting protein functions. For instance, human Dicer interacts with PKR via its helicase domain and modulates its function during SINV infection. LGP2 is also interacting with the Dicer helicase domain and inhibits Dicer catalytic activity. **(D)** In mammalian cells, RLRs can be involved in steric obstruction of viral dsRNA. Thus, RIG-I can block the binding and catalytic action of viral proteins (polymerase or capsid) or displace them (NS1), thereby allowing recognition of the dsRNA by other antiviral proteins. dsRNA, double-stranded RNA; ESC, embryonic stem cell; HBV, hepatitis B virus; IAV, influenza A virus; PKR, protein kinase R; RLR, RIG-I-like receptor; RNAi, RNA interference; SARS-CoV-2, Severe Acute Respiratory Syndrome Coronavirus 2; SINV, Sindbis virus; siRNA, small interfering RNA; vDNA, viral DNA.

There are at least 2 dmDicer-2–dependent pathways that do not rely on its canonical catalytic activity. First, both in *Drosophila* and mosquitoes, studies on persistent viral infection by RNA viruses led to the discovery of a new viral genomic element in host-infected cells [93,94]. After vsiRNA biogenesis, a transposon-encoded cellular retrotranscriptase can amplify and convert viral RNA into DNA (vDNA) [93]. In both insects, vDNA was retrieved either in linear or in circular forms (cvDNA). cvDNA are homologous to defective viral genome (DVG) and can amplify the RNAi response in insects (Fig 5A, right part). Those cvDNA are sufficient to trigger biogenesis of new siRNAs targeting the viral genome. Although the molecular mechanism has not been elucidated, vDNA synthesis is entirely dependent on dmDicer-2 DEAD-Box helicase domain, which would make it an insect RLR [93]. Indeed, this is reminiscent of the situation in mammalian cells, where DVGs are recognized by cytosolic RLRs and trigger IFN-I responses against arboviruses [95,96]. Second, dmDicer-2 helicase domain is also involved in another aspect of an IFN-like pathway, namely the capacity to induce cytokine production after detection of PAMPs. Although there is no equivalent to mammalian IFN α/ß cytokines in *Drosophila*, its genome encodes for a cysteine-rich protein named Vago, which may be considered as a cytokine-like element [67]. Upon DCV infection, Vago expression is increased and exerts antiviral effects against DCV in a manner depending on dmDicer-2 DEAD-Box helicase integrity (Fig 5A, left part). Vago expression is totally independent of other RNAi pathway members, but when dmDicer-2 helicase domain is mutated, Vago expression is dramatically decreased [67]. However, to date, a little is known regarding the pathway involved upon Vago induction by dmDicer-2 or its role in other viral infections.

The relevance of RNAi as an antiviral defense mechanism in mammals is still a matter of debate, and many reports have tried to answer the issue pertaining to the evolution of Dicer antiviral activity in metazoans [9,14,17,97]. VsiRNAs were found to be functional and to reduce viral load in a sequence-dependent fashion in undifferentiated mouse embryonic stem cells (ESCs) (Fig 5B) and somatic cells [98–100]. In oocytes, ESCs and generally, undifferentiated cells, IFN-I is not set up yet to avoid inflammatory response and apoptosis during development [101–103]. In MAVS- or IFNAR-deficient mouse somatic cells, sequence-specific hsDicer- and Ago2-dependent siRNAs are detected when cells are transfected with an exogenous long dsRNA [104]. Moreover, AGO4 was recently showed to be involved in antiviral RNAi against influenza A virus (IAV), VSV, and EMCV in mouse macrophages [105]. It seems also that viral suppressors of RNAi (VSRs) prevent Dicer from playing an antiviral role. Indeed, in mammalian cells, a decrease in viral replication was observed with flaviviruses deleted from their VSR [106].

On the other hand, other experiments on the detection of vsiRNA were not conclusive enough to validate a functional antiviral RNAi. Indeed, hsDicer is less processive than *Drosophila* dmDicer-2 to generate siRNAs from long-dsRNA [107,108]. This could explain the difficulty to detect vsiRNA in mammalian somatic cells infected with several viruses [109]. In agreement with this hypothesis, in IFN-I–deficient human cells that express the regular Dicer protein, no vsiRNA was detected when infected by SINV, Yellow fever virus (YFV), or EMCV [110]. Besides, when hsDicer is genetically invalidated, no replication increase could be observed for many RNA viruses [111].

Explanations on this defective RNAi pathway in mammals are now centered on the helicase domain of Dicer. First, as opposed to dmDicer-2, hsDicer is not processive due to the fact that it cannot hydrolyze ATP [30,59,108,112,113]. Dicer helicase domain appears to limit its functionality. Indeed, *in vitro* studies on hsDicer revealed that siRNA production is performed less efficiently than pre-miRNA to miRNA maturation [30,114]. Accordingly, in human somatic cells, only the artificial expression of a helicase-truncated form of human Dicer, named Dicer-N1, could produce functional siRNA from IAV genome with a moderate antiviral effect [115] (Fig 5B). This helicase deletion was inspired from the specific case of murine oocytes, which express a truncated version of Dicer, Dicer^O^, due to an insertion of a retrotransposon in the 5′ part of the Dicer gene [116]. The part of the helicase domain missing in Dicer^O^ is the same one that was deleted in Dicer-N1, and indeed the shorter mouse Dicer lacking part of its helicase domain has enhanced capacities in term of siRNA production from endogenous and exogenous hairpin [116]. However, the role of Dicer^O^ in antiviral defense has not been explored to date. Interestingly enough, another Dicer isoform has been recently identified in human stem cells. It also presents a deletion in its helicase domain; more precisely, it lacks the HEL2i subdomain. This splicing isoform, which has been coined Avid (for antiviral Dicer) shows enhanced antiviral RNAi properties against several RNA viruses including ZIKV and the Severe Acute Respiratory Syndrome Coronavirus 2 (SARS-CoV-2) (Fig 5B) [117].

Another possible and nonmutually exclusive explanation for the inhibition of Dicer-dependent RNAi in mammalian cells could be its interaction with a protein with repressive activity. Such a protein could be LGP2, which was shown to associate with Dicer to inhibit dsRNA cleavage (Fig 5C, right) [118]. One hypothesis to explain this observation is that there is a need to prevent dsRNA degradation by Dicer and preserve it for sensing by other DRAs, thereby suggesting a competition for substrate binding. This may be the main reason why there seems to be a crosstalk between RNAi and IFN-I. As LGP2 is similarly involved in MDA5 oligomerization enhancement, LGP2 might also bring dsRNA substrate to MDA5. Interestingly, the helicase domain of LGP2 interacts with Dicer and several other proteins involved in antiviral defense pathways including TRBP, PACT, and the dsRNA-activated protein kinase R (PKR) [118].

Finally, Dicer itself might be involved in modulating the activity of other key antiviral proteins. Dicer is already known to be involved in several interactions with cofactors via its helicase domain. Thus, 2 proteins that are known for their interaction with PKR interact with Dicer during miRNA biogenesis: TRBP and PACT [33,119]. Upon HIV-I infection, TRBP appears to bind the TAR RNA to inhibit PKR activation, thereby preventing an efficient antiviral response. PACT, another dsRNA-binding protein, is also an activator of PKR upon stress except when it interacts with TRBP [120–122]. Recently, our laboratory showed that Dicer is part of a complex involving not only TRBP and PACT but also PKR. The direct interaction of Dicer with PKR was specifically observed in cells infected with SINV and had a negative effect on PKR antiviral activity (Fig 5C, left) [123]. Another indication of the crosstalk between Dicer and PKR was reported in mouse ESCs, where Dicer represses the IFN response as well as PKR activity upon dsRNA transfection [124]. As mentioned before, Dicer could interact with ISGs but its role in the IFN-I response is not clear yet. Thus, other ISGs such as the deaminase ADAR1 or the RNA helicase DHX9 are part of the RISC assembly complex [125,126] but are also found specifically enriched within a Dicer-containing complex upon SINV infection [123]. This indicates that the helicase domain of Dicer could be involved in modulating the IFN response by acting as a central interaction platform during viral infection.

RLRs as well can be involved in IFN-independent antiviral functions (Fig 5D). Upon HBV infection, RIG-I recognizes the 5′ extremities of pre-genomic RNA and interferes with the retrotranscriptase activity [127]. Upon IAV infection, RIG-I competes with capsid molecules for the genomic RNA. Besides, both RIG-I and MDA5 can, in an ATP-dependent fashion, displace viral proteins from dsRNA including IAV NS1 [128]. Thus, this helicase-dependent activity promotes dsRNA recognition by other antiviral proteins including PKR to enhance the antiviral response [129]. Recently, RIG-I was also shown to be involved in an IFN-I independent regulation of SARS-CoV-2 infection. It recognizes the 3′ untranslated region of the SARS-CoV-2 RNA genome via the helicase domain, but not the CTD, and does not activate MAVS-dependent pathways. Rather, it seems to prevent the viral RdRp from initiating replication [130].

## Conclusions

DExD/H-box helicases are increasingly being recognized as key components of the innate antiviral response in eukaryotes. Although they all share as a core component a helicase domain able to bind dsRNA, they diverge into 2 main families characterized by their structural organization and their mode of action. Indeed, they can act either in a catalytic mode, or by triggering an antiviral signaling cascade, ultimately leading to an immune response. Here, we mainly focused on a subset of these RNA helicases, which either participate in RNAi or in IFN-I response. However, the boundary between the 2 types of response has become blurred in the past years, while there is more and more evidence that they are rather interconnected. Although Dicer is retrieved in almost all metazoans, its exact contribution to antiviral responses varies from one organism to another. Dicer has a clearly defined catalytic activity against long dsRNA in *Drosophila* and *C*. *elegans* but appears to be more limited in mammalian cells. In the latter case, this restriction seems to be mediated by the helicase domain, since its complete or partial deletion results in a more potent antiviral activity. The recent discovery of a naturally occurring splicing isoform of Dicer in stem cells, lacking the Hel2i helical subdomain, is exciting and will likely reignite the interest in the field. In addition, Dicer could have other roles during viral infection independent on dsRNA processing into siRNAs, such as the regulation of antiviral proteins like PKR. So, we clearly do not have yet a complete picture of its importance in different contexts. The tight regulation of mammalian Dicer activity is similar to what can be observed for RIG-I and MDA5, which are both regulated by posttranslational modifications or by the binding of proteins that can activate or inhibit their recognition as well as their catalytic activities [66]. Even when the situation seems to be simple at first glance, such as antiviral RNAi in *C*. *elegans*, other RNA helicases (DRH-1 to 3) were shown to be essential to maintain a robust response and help Dicer to function properly. It goes without saying that since helicases are the main immunity receptors, viruses developed counter mechanisms to block dsRNA recognition or their catalytic function [131], an interesting aspect that would require a review of its own.

In addition to the few helicases we discussed in this review, there is a large variety of other DExD/H helicases that can be involved in antiviral defense (see [132] for a recent review). In some cases, these proteins contribute to the RLRs function, but they also have the capacity to function independently of the IFN response. Some of these helicases can also have a proviral role in specific cases [133], an aspect that we did not develop here for the sake of brevity. It would be especially interesting to decipher the full involvement of these RNA helicases in modulating Dicer activity during viral infection either by modulating its ability to interact with dsRNA or its processivity. Our recent determination of the Dicer interactome upon SINV infection provides hints that this might be the case, but we need to expand the field of investigation by looking into different cell types as well as viruses. Only then will we be able to fully grasp the importance of RNA helicases in the cellular response to viral infections.
